# Mapping advanced and metastatic cancer survivorship research in Australia: a systematic scoping review

**DOI:** 10.1007/s11764-025-01937-8

**Published:** 2026-01-09

**Authors:** Andrea L. Smith, Philip Ly, Holly Keogh, Isabel Rewais, Molly Valencour, Qian Wang, Nicolas H. Hart, Carolyn Mazariego, Larissa Nekhlyudov

**Affiliations:** 1 The Daffodil Centre, The University of Sydney, and Cancer Council NSW, Sydney, NSW Australia; 2 Sydney School of Public Health, The University of Sydney, Sydney, NSW Australia; 3 Human Performance Research Centre, INSIGHT Research Institute, Faculty of Health, University of Technology Sydney, Sydney, NSW Australia; 4 Caring Futures Institute, College of Nursing and Health Sciences, Flinders University, Adelaide, SA Australia; 5 School of Population Health, University of New South Wales, Randwick, NSW Australia; 6 Department of Medicine, Brigham and Women’s Hospital, Harvard Medical School, Boston, MA USA

**Keywords:** Cancer survivorship, Advanced cancer, Metastatic cancer, Metastatic survivorship, Australia

## Abstract

**Purpose:**

To map advanced and metastatic cancer survivorship research in Australia and characterize studies according to the priority research themes outlined by the US National Cancer Institute (NCI).

**Methods:**

Systematic scoping review. MEDLINE, PsycINFO, Embase and CINAHL were searched from inception to December 2024 for cancer survivorship studies conducted in Australia, with studies reporting data for people with advanced or metastatic cancer classified using NCI’s priority research themes.

**Results:**

We identified 483 studies, of which 72 (14.9%) recruited and reported data for participants with advanced or metastatic cancer, focusing on: psychosocial research (51.4%); healthcare delivery (16.7%); health behaviors (12.5%); symptom management (11.1%); and epidemiology and surveillance (8.3%). Most were conducted within individual Australian states (72.2%) and involved multiple cancer types (41.7%). Study designs included qualitative (44.4%), cross-sectional (34.7%), cohort (16.7%), case–control (1.4%), case report (1.4%) and mixed methods (1.4%). Forty-two tumor-specific studies were identified including breast (33.3%), hematological (21.4%); prostate (11.9%); bowel/colorectal (11.9%), gynecological (9.5%), melanoma (7.1%), head and neck (2.4%), and lung (2.4%) cancers. Nearly a quarter of studies recruited participants via cancer registries (22.2%).

**Conclusions:**

Few cancer survivorship studies included people with advanced or metastatic cancer in Australia. We offer six recommendations to support funders, clinicians, policymakers, and researchers to improve representation of advanced or metastatic cancer survivors in survivorship research programs. Similar efforts in other countries are needed.

**Implications for Cancer Survivors:**

Increasing numbers of people are living long-term with advanced or metastatic cancer. The purposeful inclusion of these people in cancer survivorship research and care programs will improve health outcomes.

**Supplementary Information:**

The online version contains supplementary material available at https://doi.org/10.1007/s11764-025-01937-8.

## Introduction

In 1986, the National Coalition for Cancer Survivorship defined a cancer survivor as anyone with cancer ‘from the time of diagnosis through the balance of his or her life’ [1]. The intent was to shift the negative framing of people with cancer as ‘victims’ to more positively frame them as ‘survivors’. One of the key objectives was to draw attention to the unrecognized and therefore unmet supportive care needs of people diagnosed with cancer in the short, medium and long-term.

Until very recently, there has been relatively little attention afforded to the survivorship needs of the growing population of people living long-term with advanced or metastatic cancer, and consequently limited focus on the development of relevant interventions and models of care for this population [2–4]. This is despite widespread recognition that people affected by advanced or metastatic cancer are an underserved population with significant and unique unmet needs [5–8].Historically, prognosis was poor, often observed in months rather than years. As such, supportive care research focus for this cohort had instead been primarily on end-of-life or palliative care. However, more recently, with improved cancer treatment and prolonged survival across many cancer types, emphasis is now being placed on advanced or metastatic cancer survivorship, and there has been acknowledgement of paucity of relevant research for this population [9, 10]. In 2022, the US National Cancer Institute (NCI) summarized evidence gaps and key opportunities in advanced and metastatic survivorship research, identifying five key research areas that warranted attention: epidemiology and surveillance, symptom management, psychosocial research, healthcare delivery, and health behaviours [9].

In Australia, cancer survivorship research is a well-established field of inquiry with a robust community of practice [11]. It is recognized as a part of the cancer care continuum by Cancer Australia and a funding category in many national research funding schemes. However, to what extent Australia’s survivorship research focuses on advanced or metastatic cancer survivorship is not known. Thus, our aim was to map advanced or metastatic cancer survivorship research that focused on patients and/or their carers in Australia and to characterize the research according to the priority research themes outlined by the US National Cancer Institute (NCI).

## Methods

### Australian survivorship research studies

This systematic scoping review followed the five framework stages of Arksey and O’Malley, with adaptations: (1) identifying the research question; (2) identifying eligible studies; (3) study selection; (4) charting the data; and (5) collating, summarizing, and reporting the results [12–14]. As this review provides an overview of evidence regardless of methodological quality or risk of bias, no quality assessment was conducted, consistent with best-practice recommendations and the Preferred Reporting Items for Systematic reviews and Meta-Analyses extension for Scoping Reviews (PRISMA-ScR) reporting guidelines [14–16]. For this review, we used Hart and colleagues’ definition of advanced and metastatic cancer, that is, advanced cancer is broadly defined as any solid or hematological malignancy unlikely to be cured or controlled with treatment, whereas metastatic cancer is defined as the de novo or recurrent diagnosis of any cancer where the primary tumor has spread beyond its origin to a distant organ or tissue [2].

### Identifying the research question

The research question was developed through discussions with cancer survivorship researchers in Australia and internationally, and observations of the type of research presented at Australian cancer survivorship conferences. The final research questions were: ‘To what extent does Australian cancer survivorship research report on those living with advanced or metastatic cancer?’ and ‘What is the research focus of these studies?’. PROSPERO, the National Institute for Health and Care Research’s international prospective register of systematic reviews, and the Cochrane library were searched yielding no similar reviews.

### Identifying eligible studies

Four electronic databases (MEDLINE, PsycINFO, Embase and CINAHL) were searched from their inception until the 2nd of December 2024. A comprehensive search strategy was developed with advice from an academic librarian with expertise in database searches (Supplementary File 1). The Population-Concept-Context (PCC) framework was used to determine the focus and context for the review: adults (≥ 18 years old) or carers of adults with cancer (= population); the study authors referred to participants as ‘cancer survivors’ or ‘cancer survivorship’ (= concept); and the study was conducted in Australia (or ≥ 30% study participants based in Australia) (= context) [14]. Given the assumption that all articles included in the *Journal of Cancer Survivorship* report survivorship research, we hand-searched all issues from its inception in 2007 until December 2024 for Australian research papers. Full eligibility criteria are provided in Table 1.

**Table 1:** Eligibility criteria

Inclusion criteria	Exclusion criteria
Population	
• Adults diagnosed with cancer • Carers of adults diagnosed with cancer	• Paediatric patients diagnosed with cancer • Adolescents and young adults (AYA) diagnosed with cancer • Carers of a paediatric/AYA diagnosed with cancer • Healthcare professionals who provide supportive care to paediatric/AYA diagnosed with cancer • Healthcare professionals
Concept	
• Cancer survivor or survivorship	
Context	
• Study participants must be based in Australia (or at least 30% of study participants are based in Australia)	
Type of article	
• Peer-reviewed research articles	• Study protocols; reviews; conference abstracts; commentaries; editorials; papers with no full text available

### Study selection

Titles, abstracts, and full texts were independently screened and reviewed by two researchers (ALS, PL, HK, IR, MV, QW or CM), with conflicts discussed and resolved by a third researcher (ALS or CM).

### Charting the data

#### Identifying papers reporting data for advanced or metastatic cancer survivors (or their carers)

**Fig. 1:**
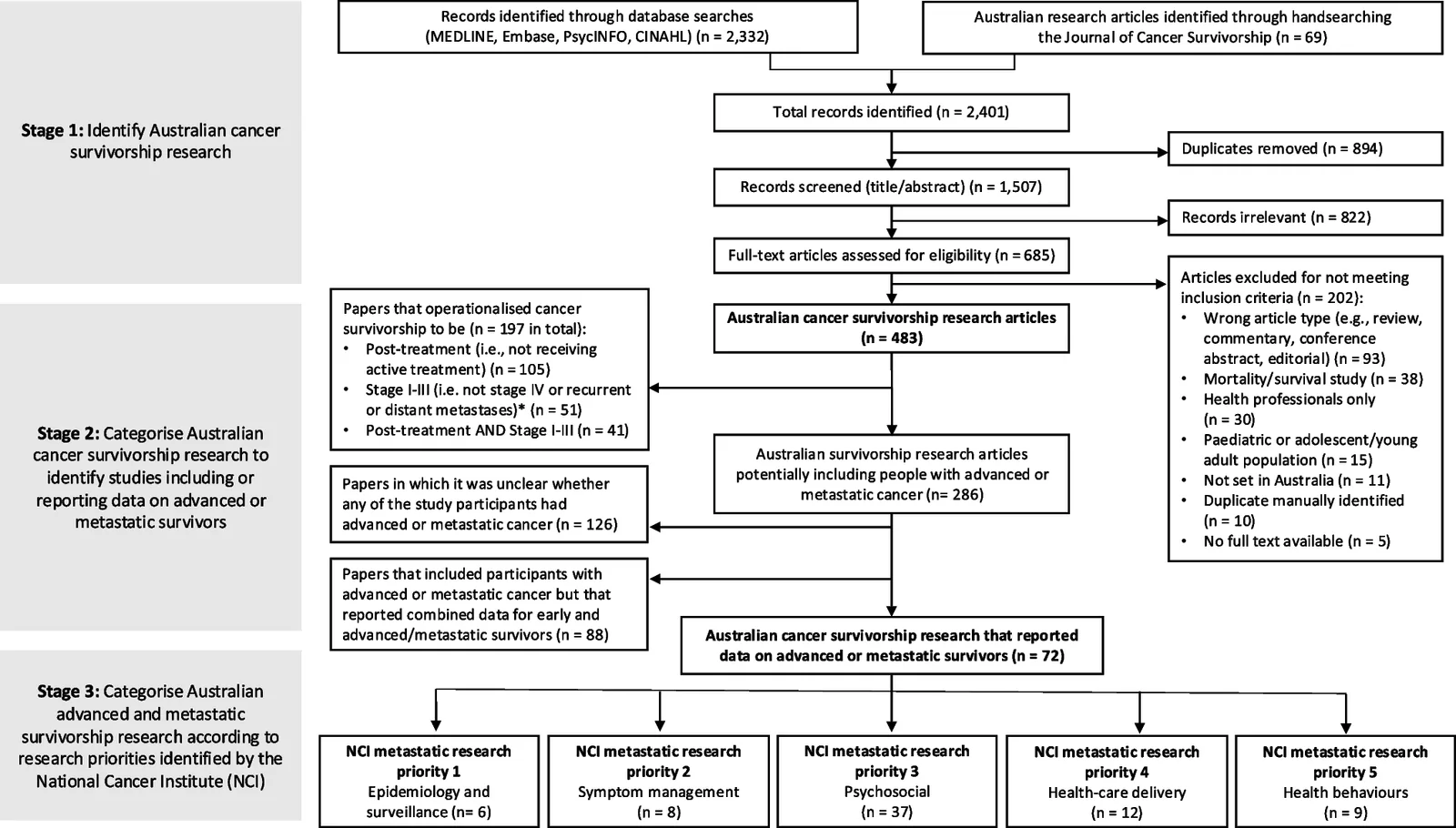
Pathway for the screening and categorization of Australian metastatic and advanced cancer survivorship research. *As the usual staging criteria used for solid tumors cannot be used to classify hematological cancers, we used the TbnC cohort profile outlined by White et al. [17]

We categorized the identified studies using a two-step process. First, studies were categorized according to how the term survivorship was operationalized and the impact on participant eligibility (Fig. 1). We sorted studies based on whether they required participants to be: (1) post-treatment (i.e., not receiving active treatment); (2) stage I-III (i.e., not stage IV, recurrent distant disease or distant metastases); or (3) post-treatment and stage I-III. Given staging varies across cancer types (e.g., carcinomas, sarcomas, myelomas, leukemias, and lymphomas), we used White’s definitions of treatable-but-not-curable (TbnC) cancer to guide how we sorted studies [17]. Second, we then categorized the survivorship research that potentially included participants who had metastatic or advanced cancer (or were carers of these people) into three categories so that we could understand what data relevant to those with advanced or metastatic cancer had been reported: (1) unclear whether any of the study participants had advanced or metastatic cancer; (2) participants with advanced or metastatic cancer were recruited but findings reported only combined data for early and advanced or metastatic participants (i.e., unable to distinguish specific data for advanced or metastatic participants); and (3) participants with advanced or metastatic cancer were recruited and data for this population were reported.

#### Extracting, mapping data for advanced or metastatic cancer survivors (or their carers)

Data were extracted from the studies that included, recruited, and reported data on people with advanced or metastatic cancer (or their carers). Data extraction was performed by one author (PL or QW) and verified by a second author (ALS) (Table 2). Finally, each study was categorized according to advanced and metastatic research priorities identified by the NCI: epidemiology and surveillance, symptom management, psychosocial research, healthcare delivery, and health behaviors (Table 3) [9].

**Table 2:** Characteristics of included studies (*n* = 72)

Characteristics of studies	Number of studies, N (%)
**Year of publication**	
2000–2004	1 (1.4)
2005–2009	5 (6.9)
2010–2014	13 (18.1)
2015–2019	20 (27.8)
2020–2024	33 (45.8)
**State/territory or national recruitment**^**a**^	
State/territory recruitment^b^	52 (72.2)
New South Wales	20 (27.8)
Queensland	15 (20.8)
Victoria	11 (15.3)
Western Australia	4 (5.6)
South Australia	2 (2.8)
Tasmania	0 (0.0)
Northern Territory	0 (0.0)
Australian Capital Territory	0 (0.0)
National	15 (20.8)
Not reported	5 (6.9)
**Proportion of study cohort who had advanced or metastatic cancer**^**c**^	
Not reported	27 (37.5)
< 10%	12 (16.7)
10–30%	9 (12.5)
31–60%	8 (11.1)
61–90%	2 (2.8)
91–100%	14 (19.4)
**Cancer type**	
Mixed	30 (41.7)
Breast	14 (19.4)
Hematological	9 (12.5)
Prostate	5 (6.9)
Bowel/colorectal	5 (6.9)
Gynaecological	4 (5.6)
Melanoma	3 (4.2)
Lung	1 (1.4)
Head and neck	1 (1.4)
**Inclusion of carers**	
Yes	12 (16.7)
No	60 (83.3)
**Cancer registry-based recruitment**	
Yes	16 (22.2)
Population-based cancer registry used	15 (20.8)
Clinical cancer registry used	1 (1.4)
No	42 (58.3)
**Study design**	
Qualitative research	32 (44.4)
Cross-sectional study	25 (34.7)
Cohort study	12 (16.7)
Case–control study	1 (1.4)
Case report	1 (1.4)
Mixed methods	1 (1.4)

^a^Four studies also recruited participants from outside of Australia

^b^In Australia, a state is a constitutionally recognised sub-national entity with its own parliament and legislative authority over key domains such as health, education, and transport. Australia comprises six states and two self-governing territories, each operating within a shared federal system

^c^In studies with a mixed population (e.g., patients, carers and health professionals), the proportion of participants with advanced or metastatic cancer relates specifically to the status of the patients only

**Table 3:** National Institute of Cancer’s advanced and metastatic research priorities (*n* = 72) [9]

	N (%)
**Research priority**	
Psychosocial research	37 (51.4)
Healthcare delivery research	12 (16.7)
Health behaviors research	9 (12.5)
Symptom management research	8 (11.1)
Epidemiology and surveillance	6 (8.3)

### Collating, summarizing and reporting the results

Descriptive analyses (counts and percentages) of the extracted data were performed and reported.

## Results

The systematic search strategy identified 2,401 studies (2,332 from database searches; 69 from hand-searching) (Fig. 1). After deduplication and screening, 483 studies were included.

### Participants with advanced or metastatic cancer and reporting of data for this population

Of 483 Australian cancer survivorship studies, 40.8% (197/483) required participants to be post-treatment (21.7%; 105/483), stage I-III (10.6%; 51/483) or both (8.5%; 41/483) (Fig. 1). The remaining 286 studies included individuals with advanced or metastatic cancer; however, in 44.1% (126/286) of studies it was unclear whether any of the recruited participants had advanced or metastatic cancer and in nearly a third (30.8%; 88/286) the study authors did not report data specific to those with advanced or metastatic cancer, instead reporting combined data for localized and advanced/metastatic cancer populations. This left 72 (14.9%; 72/483) Australian cancer survivorship studies that reported data specifically relating to the advanced or metastatic cancer survivor population. Categorization of these 72 studies according to the NCI research priorities indicated that most reported on psychosocial research (51.4%; 37/72), followed by healthcare delivery (16.7%; 12/72), health behaviors (12.5%; 9/72), symptom management (11.1%; 8/72) and epidemiology and surveillance (8.3%; 6/72) (Table 3).

### Study and participant characteristics in studies including people with advanced or metastatic cancers

Characteristics of the 72 included studies are summarized in Table 2 (see Supplementary File 2 for further study details). Nearly half of the studies used a qualitative methodology (44.4%; 32/72), with the remainder using cross-sectional (34.7%; 25/72), cohort (16.7%; 12/72), case–control (1.4%; 1/72), case report (1.4%; 1/72) or mixed methods (1.4%; 1/72).

Most of the studies reported on research that was state-based (i.e. regional rather than national) (72.2%; 52/72) and many of the studies examined multiple cancer types (41.7%; 30/72). There were 42 tumor-specific studies, of which: 33.3% (14/42) were breast; 21.4% (9/42) hematological; 11.9% (5/42) prostate; 11.9% (5/42) bowel/colorectal, 9.5% (4/42) gynecological, 7.1% (3/42) melanoma, 2.4% (1/42) head and neck, and 2.4% (1/42) lung cancers. Notably, about a quarter of studies recruited participants through cancer registries (22.2%; 16/72), making this one of the more common, though not predominant, recruitment methods. Of these, 93.8% (15/16) were from population-based registries and 6.3% (1/16) from clinical registries.

Over a third (37.5%; 27/72) of the studies that included people with advanced or metastatic cancer did not report the number or percentage (Table 2; Supplementary file 2). Of the 45 studies (62.5%; 45/72) that reported the percentage of participants with advanced or metastatic cancer, 21/45 (46.7%) included ≤ 30.0%, 8/45 (17.8%) included 31–60%, 2/45 (4.4%) included 61–90%, and 14/45 (31.1%) included 91–100%. Of the 14 studies that included a large proportion of participants with advanced or metastatic cancer (91–100%), 12 (85.7%) were qualitative.

## Discussion

Based on our scoping review of cancer survivorship research in Australia we make several observations. First, cancer survivorship studies did not typically include people affected by advanced or metastatic cancer. If these people were included, they usually comprised a small proportion of the overall study cohort, limiting the capacity to draw conclusions specific to their needs. Second, sub-group analyses often combined participants with localized and metastatic cancer, which again may limit our understanding of specific metastatic survivorship care needs and the relevance, acceptability and impact of survivorship interventions on those with metastatic cancer. Third, in studies reporting advanced and metastatic survivorship data, the most common research focus was on the psychological, emotional and social aspects of survivorship. In contrast, there were a limited number of studies with an epidemiological or surveillance focus. Given epidemiological studies play an important role in understanding disease patterns, treatment outcomes, and long-term health impacts, this highlights an important research gap. Our findings are consistent with prior literature identifying paucity in research and opportunities, as well as calls for targeted funding for advanced and metastatic survivorship research [10, 18–20].

Novel cancer treatments are driving increased post-metastatic survival of people diagnosed with certain types of metastatic cancer (e.g., multiple myeloma, breast, prostate, lung, melanoma, colorectal), resulting in the emergence of a growing population of people needing long-term survivorship support (and possibly palliative care) before ultimately requiring end-of-life care [4, 9, 17, 21]. Further, while survivorship research has traditionally focused on individuals treated with curative intent who have completed their primary treatment, there is now a growing recognition that many people with an advanced or metastatic diagnosis, while not likely to be cured, are also not at the end-of-life stage but are instead occupying the ‘place in between’ cure and death [22]. The treatment intent is not curative nor end-of-life care; the intent is instead to prolong life while maintaining quality of life for as long as possible [9]. As such, focus on advanced and metastatic survivorship has grown and it is imperative that it continues to evolve. There are some methodological challenges in including people with advanced or metastatic cancer in survivorship studies. Our review indicates that the ability to identify people with advanced or metastatic cancer in cancer and health data sets is a critical issue [9]. For example, many national cancer surveys do not include questions that would enable identification of important subgroups of survivors, such as groups based on stage or metastatic/recurrence status [23]. Further, population-based registries likewise do not capture recurrence or cancer progression [21]. Consequently, unless study participants recruited via cancer registries are contacted and asked about recurrence or disease progression, any relationships reported between cancer-registry determined stage (i.e. stage I-III versus metastatic cancer) and variables such as treatment side-effect burden, psychosocial effects, financial toxicity, and return to work may be inaccurate. For researchers to be able to identify and recruit participants with an advanced or metastatic diagnosis, there must be focused efforts to improve cancer registry collection and reporting of stage at diagnosis and recurrence data. Alternatively, innovative solutions such as linkage of health record data sets potentially provide a means of identifying individuals with advanced or metastatic disease in the absence of recurrence data [21].

A further methodological challenge is recruitment and retention of individuals with advanced or metastatic cancer owing to issue relating to symptom burden, disease progression and/or mortality.

### Recommendations

This review underscores a significant gap in cancer survivorship research: the limited inclusion and visibility of individuals living with advanced or metastatic cancer. While some studies included this population, few provided disaggregated data or tailored insights. To address this, we propose five key recommendations that aim to improve transparency in reporting, strengthen methodological rigour, and enhance the relevance of survivorship research for people with advanced or metastatic disease (Table 4). These recommendations advocate for clearer definitions of survivorship populations, justification for inclusion criteria, acknowledgment of design limitations, improved data infrastructure, and intentional oversampling of advanced and metastatic cohorts. Collectively, they offer a roadmap for more inclusive and representative research, with the potential to inform policy, funding, and clinical practice that better reflects the needs of people with advanced or metastatic cancer.

**Table 4:** Recommendations

**Recommendation 1: Clear reporting of the survivorship population**
• State in the article, ideally in title or abstract, whether the study’s survivorship cohort comprises individuals with early-stage cancer (i.e. post-treatment and/or stage I-III), early-stage and advanced or metastatic cancer, or advanced and metastatic cancer only
• Provide a definition of ‘survivor’ in the context of the study
**Recommendation 2: Provide rationale for the survivorship population being studied**
• Depending on the study design, research question and aims, it may not be possible or appropriate to include individuals with advanced or metastatic cancer. If recruitment is limited to post-treatment and/or stage I-III survivors, provide a rationale for why this decision was made. For example, researchers could report whether limiting inclusion to those post-treatment and/or stage I-III is due to the nature of the intervention or research question, or if it is a methodological constraint such as potential difficulties recruiting a sufficient sample size
**Recommendation 3: Critically examine and acknowledge limitations in the survivorship study design that may impact on the accuracy, generalisability or relevance of the findings**
• If the research includes advanced or metastatic survivors but a limited number of advanced or metastatic participants were recruited, acknowledge this as a limitation and if necessary, note that there were insufficient data to report findings for this population
• Researchers should consider how the design of their studies, including recruitment, data collection and analysis, may influence the generalisability of their findings to the wider cancer population. This may require a better understanding of the limitations of the database from which researchers are drawing their study population. In particular, claims that samples are representative of the general cancer population should be considered carefully. For example:
◦ if recruiting or using data from cancer registries, it is essential to acknowledge the inherent limitation given that such methods will typically not identify individuals with recurrent metastatic cancer and sometimes do not systematically identify those who are stage IV at diagnosis
◦ ensure sufficient recruitment to allow subgroup analyses of advanced or metastatic cohorts. If the study does not include cancer stage in the analysis (e.g. analysis not possible as groups are too small), then a sensitivity analysis could be performed to show that there is no difference from the analysis with/without stage data
**Recommendation 4: Develop methods to identify survivors diagnosed with or who have progressed to advanced or metastatic cancer**
• Population-based cancer registries and other large databases should ideally capture stage at diagnosis and recurrence data so that: ◦ participants have their advanced or metastatic status correctly reported in research studies that draw on these databases ◦ advanced and metastatic incidence and prevalence can be reported, which would highlight the size of this population and potentially drive advanced or metastatic survivorship funding and research
**Recommendation 5: Oversample individuals with advanced or metastatic cancer**
• Recruiting and retaining participants with advanced or metastatic cancer in research studies can be challenging. This may be due to several factors, including the physical and emotional burden of treatment, and the higher likelihood of mortality compared to survivors with early-stage cancer. Advanced and metastatic cancer survivors may need to be oversampled in order to produce adequate data for this population

### Limitations

While this is the first study to investigate the proportion and type of cancer survivorship research relevant to advanced and metastatic cancers in Australia, we must also acknowledge some limitations. First, although our search focused on Australian research, the methodology could be applied to any country. Second, we may not have captured the entirety of the Australian cancer survivorship research but mainly research that self-identified as survivorship research via descriptors. Other relevant advanced and metastatic survivorship studies (e.g. supportive care in metastatic and advanced cancer patients; or palliative care studies) may have been excluded if they did not explicitly use the terms ‘survivor’ or ‘survivorship’. Third, while we used the treatable but not curable cohort profile as defined by White and colleagues [17], it remains challenging to classify certain advanced cancers such as hematological cancers and brain cancers. Fourth, our selection criteria may have resulted in qualitative studies being over-represented in our review. Qualitative studies exploring the lived experience of cancer survivorship often had quite general inclusion criteria, that is, they did not explicitly limit recruitment to those post-treatment or stage I-III. In addition, the use of quotes and narrative data meant qualitative studies often fulfilled our inclusion criteria of reporting data for people with metastatic cancer, although often only very limited data were reported. Finally, our review was limited to selected study characteristics and thus may not capture the settings and contexts in which cancer survivorship research in this population is conducted.

## Conclusion

Cancer survivorship research in Australia has to date overwhelmingly focused on individuals with early stage or curable cancer. With the emergence of a growing population of people living long term with an advanced or metastatic diagnosis, there is an opportunity to expand survivorship studies to include these individuals or to design studies specific to the needs of those living with advanced or metastatic cancer. Targeted research will help to develop the evidence base necessary to inform development of interventions and models of care for people with advanced or metastatic cancer, to monitor outcomes and to understand disparities.

## Supplementary Information

Below is the link to the electronic supplementary material.

Supplementary file1 (DOCX 254 KB)

Supplementary file2 (DOCX 130 KB)

## Data Availability

The data used to support the findings of this study are available from the corresponding author upon request.

## Abbreviations

*TbnC*: Treatable but not curable
*NCI*: National Cancer Institute
