# Supplementary material for: Mapping advanced and metastatic cancer survivorship research in Australia: a systematic scoping review
Source: J Cancer Surviv. 2026 Jan 9;20(Suppl 2):2251–8. doi: 10.1007/s11764-025-01937-8 (PMC13744563; doi:10.1007/s11764-025-01937-8)
Supplement: Supplementary file 1 [file 11764_2025_1937_MOESM1_ESM.docx]

**Medline (via OVID)**

**Embase (via OVID)**

**PsychINFO (via OVID)**

**CINAHL**
