# Supplementary material for: Mapping advanced and metastatic cancer survivorship research in Australia: a systematic scoping review
Source: J Cancer Surviv. 2026 Jan 9;20(Suppl 2):2251–8. doi: 10.1007/s11764-025-01937-8 (PMC13744563; doi:10.1007/s11764-025-01937-8)
Supplement: Supplementary file 2 [file 11764_2025_1937_MOESM2_ESM.docx]

Supplementary table with individual study characteristics and research priorities

**Supplementary Table 1. Individual study characteristics and research priorities**

| **Author** | **Year** | **Study design** | **State or National** | **Participants (N)** | **Participants with advanced or metastatic cancer**  **(N (%))**** | **Cancer type** | **Inclusion of carers (Y/N)** | **Research priority** |
| --- | --- | --- | --- | --- | --- | --- | --- | --- |
| Alananzeh et al.(1) | 2019 | Cross-sectional study | State (NSW)* | 143* | Unclear/Not reported | Mixed | N | Psychosocial |
| Austin et al.(2) | 2010 | Cross-sectional study | State (NSW) | 44 | 44 (100.0) | Bowel/Colorectal | N | Psychosocial |
| Baggio et al.(3) | 2024 | Cross-sectional study | National | 111 | 111 (100.0) | Leukemia | N | Epidemiology and surveillance |
| Beamont et al.(4) | 2022 | Case report | State (VIC) | 1 | 1 (100.0) | Breast | N | Symptom management |
| Beatty et al.(5) | 2021 | Qualitative research | State (SA) | 8 | 8 (100.0) | Breast | N | Health-care delivery |
| Beesley et al.(6) | 2008 | Cross-sectional study | State (QLD) | 775 | 183 (23.6) | Gynaecological | N | Health behaviors |
| Bell et al.(7) | 2013 | Cohort study | State (VIC) | 1,588 | Unclear/Not reported | Breast | N | Symptom management |
| Boyes et al.(8) | 2009 | Cross-sectional study | State (NSW) | 863 | 149 (19.8) | Mixed | N | Psychosocial |
| Broom et al.(9) | 2019 | Qualitative research | State (QLD) | 80 | Unclear/Not reported | Mixed | Y | Psychosocial |
| Bryant et al.(10) | 2020 | Cross-sectional study | State (NSW) | 144 | 47 (34.0) | Mixed | N | Health-care delivery |
| Butow et al.(11) | 2013 | Cross-sectional study | National | 596 | 16 (3.0) | Mixed | N | Psychosocial |
| Campbell et al.(12) | 2024 | Qualitative research | National | 644 | Unclear/Not reported | Breast | N | Psychosocial |
| Daniels et al.(13) | 2024 | Cohort study | State (NSW) | 21,843 | 9,802 (44.9) | Mixed | N | Health-care delivery |
| deRooij et al.(14) | 2022 | Cohort study | State (WA) | 265 | 16 (6.0) | Breast | N | Symptom management |
| Dhillon et al.(15) | 2018 | Cohort study | State (NSW)* | 434* | 73 (16.8) | Bowel/Colorectal | N | Symptom management |
| Douglas et al.(16) | 2023 | Cross-sectional study | State (NSW) | 27 | 5 (18.5) | Head & Neck | N | Psychosocial |
| Dumalaon-Canaria et al.(17) | 2018 | Cross-sectional study | National | 314 | 39 (8.2) | Breast | N | Psychosocial |
| Dunn et al.(18) | 2018 | Qualitative research | National | 21 | Unclear/Not reported | Prostate | Y | Psychosocial |
| Dunn et al.(19) | 2013 | Cohort study | State (QLD) | 1,966 | Unclear/Not reported | Bowel/Colorectal | N | Psychosocial |
| Edge et al.(20) | 2021 | Qualitative research | Unclear | 496 | 126 (25.4) | Mixed | Y | Psychosocial |
| Ee et al.(21) | 2019 | Cross-sectional study | National | 309 | 14 (4.6) | Breast | N | Health behaviors |
| Fleming et al.(22) | 2020 | Cohort study | Unclear | 408 | 27 (6.6) | Gynaecological | N | Health behaviors |
| Garvey et al.(23) | 2018 | Qualitative research | State (QLD) | 155 | 31 (20.0) | Mixed | N | Psychosocial |
| Garvey et al.(24) | 2016 | Cohort study | State (QLD) | 155 | 31 (20.0) | Mixed | N | Psychosocial |
| Gillies et al.(25) | 2023 | Cross-sectional study | State (VIC) | 46 | 2 (4.2) | Breast | N | Symptom management |
| Hall et al.(26) | 2016 | Qualitative research | State (NSW) | 259 | Unclear/Not reported | Haematological | N | Health behaviors |
| Humpel et al.(27) | 2006 | Qualitative research | State (NSW) | 19 | Unclear/Not reported | Mixed | N | Health behaviors |
| Johnston et al.(28) | 2022 | Cross-sectional study | National | 286 | 105 (36.7) | Ovarian | N | Health behaviors |
| Jones et al.(29) | 2000 | Qualitative research | Unclear | 7 | 7 (100.0) | Mixed | N | Psychosocial |
| Joshy et al.(30) | 2023 | Cohort study | State (NSW) | 15,570 | 391 (0.0) | Mixed | N | Symptom management |
| Kirby et al.(31) | 2022 | Qualitative research | State (QLD) | 190 | Unclear/Not reported | Mixed | Y | Psychosocial |
| Kirby et al.(32) | 2020 | Qualitative research | State (QLD) | 80 | 53 (66.3) | Mixed | N | Psychosocial |
| Lai-Kwon et al.(33) | 2019 | Cross-sectional study | State (VIC) | 105 | 105 (100.0) | Melanoma | N | Symptom management |
| Lai-Kwon et al.(34) | 2021 | Qualitative research | State (VIC) | 20 | 20 (100.0) | Lung | N | Health-care delivery |
| Lewis et al.(35) | 2020 | Qualitative research | State (QLD) | 50 | 39 (78.0) | Mixed | Y | Psychosocial |
| Lewis et al.(36) | 2024 | Qualitative research | National | 38 | 38 (100.0) | Breast | N | Psychosocial |
| Lim et al.(37) | 2023 | Qualitative research | State (NSW) | 28 | 38 (100.0) | Colorectal | N | Health-care delivery |
| Liu et al.(38) | 2023 | Qualitative research | State (QLD) | 16 | 7 (43.8) | Prostate | N | Psychosocial |
| Lisy et al.(39) | 2020 | Cross-sectional study | State (VIC) | 476 | Unclear/Not reported | Melanoma | N | Psychosocial |
| Lynagh et al.(40) | 2015 | Cross-sectional study | National | 431 | Unclear/Not reported | Haematological | N | Health behaviors |
| Lynch et al.(41) | 2020 | Cross-sectional study | State (VIC) | 61 | 61 (100.0) | Melanoma | N | Health-care delivery |
| McGrath(42) | 2014 | Qualitative research | State (QLD) | 50 | Unclear/Not reported | Haematological | N | Psychosocial |
| McGrath et al.(43) | 2014 | Qualitative research | State (QLD) | 50 | Unclear/Not reported | Haematological | N | Psychosocial |
| McGrath et al.(44) | 2011 | Qualitative research | State (WA) | 9 | Unclear/Not reported | Mixed | N | Psychosocial |
| Morris et al.(45) | 2011 | Qualitative research | Unclear* | 27* | Unclear/Not reported | Breast | N | Psychosocial |
| [Muhandiramge](https://pubmed.ncbi.nlm.nih.gov/?term=Muhandiramge+J&cauthor_id=39308168) et al.(46) | 2024 | Cross-sectional study | National* | 1,392 | 269 (19.3) | Mixed | N | Epidemiology and surveillance |
| Ng et al.(47) | 2020 | Case control study | National | 26,741 | Unclear/Not reported | Mixed | N | Health-care delivery |
| O'Shaughnessy et al.(48) | 2015 | Qualitative research | State (SA) | 30 | Unclear/Not reported | Prostate | Y | Psychosocial |
| Oberoi et al.(49) | 2017 | Cohort study | State (VIC) | 414 | Unclear/Not reported | Haematological | N | Psychosocial |
| Parton et al.(50) | 2019 | Mixed | Unclear | 878 | Unclear/Not reported | Mixed | Y | Psychosocial |
| Pascal et al.(51) | 2010 | Qualitative research | State (VIC) | 15 | Unclear/Not reported | Mixed | N | Psychosocial |
| Pascal et al.(52) | 2016 | Qualitative research | State (VIC) | 19 | Unclear/Not reported | Mixed | N | Psychosocial |
| Paul et al.(53) | 2013 | Cross-sectional study | State (WA) | 268 | Unclear/Not reported | Haematological | N | Health-care delivery |
| Plage(54) | 2021 | Qualitative research | State (QLD) | 12 | 4 (33.3) | Mixed | Y | Psychosocial |
| Plage(55) | 2020 | Qualitative research | State (QLD) | 11 | 4 (36.3) | Mixed | N | Psychosocial |
| Plage et al.(56) | 2022 | Qualitative research | State (QLD) | 11 | 4 (36.3) | Mixed | N | Psychosocial |
| Player et al.(57) | 2014 | Qualitative research | State (NSW) | 9 | 1 (11.1) | Breast | N | Symptom management |
| Prior(58) | 2009 | Qualitative research | State (QLD) | 13 | Unclear/Not reported | Mixed | Y | Health-care delivery |
| Pye et al.(59) | 2023 | Qualitative research | State (NSW) | 7 | Unclear/Not reported | Mixed | N | Health-care delivery |
| Sharpe et al.(60) | 2005 | Cross-sectional study | State (NSW) | 142 | 142 (100.0) | Mixed | Y | Health-care delivery |
| Shaw et al.(61) | 2018 | Cross-sectional study | National* | 152* | 4 (4.4) | Breast | N | Psychosocial |
| Skandarajah et al.(62) | 2021 | Cross-sectional study | State (VIC) | 459 | 5 (1.1) | Breast | N | Epidemiology and surveillance |
| Smith et al.(63) | 2018 | Cohort study | State (NSW) | 51,924 | Unclear/Not reported | Prostate | N | Epidemiology and surveillance |
| Stephens et al.(64) | 2018 | Qualitative research | National | 14 | Unclear/Not reported | Mixed | N | Psychosocial |
| Stephens et al.(65) | 2014 | Qualitative research | State (NSW) | 21 | 21 (100.0) | Haematological | Y | Psychosocial |
| Thandrayen et al.(66) | 2022 | Cross-sectional study | State (NSW) | 8,333 | 333 (4.0) | Mixed | N | Epidemiology and surveillance |
| Tran et al.(67) | 2020 | Cross-sectional study | State (NSW) | 20,811 | Unclear/Not reported | Mixed | N | Epidemiology and surveillance |
| Tzelepis et al.(68) | 2018 | Cross-sectional study | National | 1,417 | Unclear/Not reported | Haematological | N | Health-care delivery |
| Webb et al.(69) | 2024 | Qualitative research | National | 24 | 8 (34.8) | Ovarian | Y | Psychosocial |
| Yee et al.(70) | 2014 | Cohort study | State (NSW) | 71 | 71 (100.0) | Breast | N | Health behaviors |
| Zhang et al.(71) | 2020 | Cohort study | State (NSW) | 2,395 | 150 (6.3) | Bowel/Colorectal | N | Psychosocial |
| Zopf et al.(72) | 2017 | Cross-sectional study | State (WA) | 55 | 55 (100.0) | Prostate | N | Health behaviors |

* Five studies also recruited participants from outside of Australia
** In studies with a mixed population (e.g., patients, carers and health professionals), the proportion of participants with advanced or metastatic cancer relates specifically to the status of the patients only

References

1.	Alananzeh IM, Levesque JV, Kwok C, Salamonson Y, Everett B. The Unmet Supportive Care Needs of Arab Australian and Arab Jordanian Cancer Survivors: An International Comparative Survey. Cancer Nursing. 2019;42(3):E51-E60.

2.	Austin KK, Young JM, Solomon MJ. Quality of life of survivors after pelvic exenteration for rectal cancer. Dis Colon Rectum. 2010;53(8):1121-6.

3.	Baggio D, Chung E, Wellard C, Waters N, Cushion T, Chong G, et al. Australians with chronic lymphocytic leukaemia continue to have high rates of second primary malignancies in the modern era. Intern Med J. 2024;54(7):1223-7.

4.	Beaumont S, Angel CM, Dawson SJ. Denosumab and invasive cervical root resorption: a case report. Aust Dent J. 2022;67(2):183-9.

5.	Beatty L, Koczwara B, Butow P, Turner J, Girgis A, Schofield P, et al. Development and usability testing of a web-based psychosocial intervention for women living with metastatic breast cancer: Finding My Way-Advanced. J Cancer Surviv. 2021;15(3):403-9.

6.	Beesley VL, Eakin EG, Janda M, Battistutta D. Gynecological cancer survivors' health behaviors and their associations with quality of life. Cancer Causes Control. 2008;19(7):775-82.

7.	Bell RJ, Robinson PJ, Barallon R, Fradkin P, Schwarz M, Davis SR. Lymphedema: experience of a cohort of women with breast cancer followed for 4 years after diagnosis in Victoria, Australia. Support Care Cancer. 2013;21(7):2017-24.

8.	Boyes AW, Girgis A, Zucca AC, Lecathelinais C. Anxiety and depression among long-term survivors of cancer in Australia: results of a population-based survey. Med J Aust. 2009;190(S7):S94-8.

9.	Broom A, Kenny K, Kirby E, Lwin Z. The collective/affective practice of cancer survivorship. Br J Sociol. 2019;70(4):1582-601.

10.	Bryant J, Turon H, Mansfield E, Cameron E, Dodd N. Discussions About Lifestyle Risk Factors Following a Cancer Diagnosis: Findings from a Sample of Australian Cancer Outpatients. J Cancer Educ. 2020;35(6):1170-6.

11.	Butow PN, Aldridge L, Bell ML, Sze M, Eisenbruch M, Jefford M, et al. Inferior health-related quality of life and psychological well-being in immigrant cancer survivors: a population-based study. Eur J Cancer. 2013;49(8):1948-56.

12.	Campbell B, Mackenzie L, Lewis J. Beyond breast cancer: An exploration of the experiences of middle-aged female breast cancer survivors in Australia. Maturitas. 2024;188:108089.

13.	Daniels B, Luckett T, Liauw W, Falster MO, Gisev N, Blyth FM, et al. Trajectories of Opioid Use Before and After Cancer Diagnosis: A Population-Based Cohort Study. J Pain Symptom Manage. 2024;68(3):282-91.e11.

14.	de Rooij BH, Ramsey I, Clouth FJ, Corsini N, Heyworth JS, Lynch BM, et al. The association of circadian parameters and the clustering of fatigue, depression, and sleep problems in breast cancer survivors: a latent class analysis. J Cancer Surviv. 2022.

15.	Dhillon HM, Tannock IF, Pond GR, Renton C, Rourke SB, Vardy JL. Perceived cognitive impairment in people with colorectal cancer who do and do not receive chemotherapy. J Cancer Surviv. 2018;12(2):178-85.

16.	Douglas C, Hewitt L, Yabe TE, Mitchell J, Ashford B. Quality of Life Impacts Following Surgery for Advanced Head and Neck Cancer. World J Oncol. 2023;14(2):150-7.

17.	Dumalaon-Canaria JA, Prichard I, Hutchinson AD, Wilson C. Fear of cancer recurrence and psychological well-being in women with breast cancer: The role of causal cancer attributions and optimism. Eur J Cancer Care (Engl). 2018;27(1).

18.	Dunn J, Casey C, Sandoe D, Hyde MK, Cheron-Sauer MC, Lowe A, et al. Advocacy, support and survivorship in prostate cancer. Eur J Cancer Care (Engl). 2018;27(2):e12644.

19.	Dunn J, Ng SK, Breitbart W, Aitken J, Youl P, Baade PD, et al. Health-related quality of life and life satisfaction in colorectal cancer survivors: trajectories of adjustment. Health Qual Life Outcomes. 2013;11:46.

20.	Edge R, Mazariego C, Li Z, Canfell K, Miller A, Koczwara B, et al. Psychosocial impact of COVID-19 on cancer patients, survivors, and carers in Australia: a real-time assessment of cancer support services. Support Care Cancer. 2021;29(9):5463-73.

21.	Ee C, Cave AE, Naidoo D, Boyages J. Prevalence of and attitudes towards complementary therapy use for weight after breast cancer in Australia: a national survey. BMC Complement Altern Med. 2019;19(1):332.

22.	Fleming S, Jones T, Janda M, Vagenas D, Ward L, Reul-Hirche H, et al. Physical activity trajectories following gynecological cancer: results from a prospective, longitudinal cohort study. Int J Gynecol Cancer. 2020;30(11):1784-90.

23.	Garvey G, Cunningham J, Janda M, Yf He V, Valery PC. Psychological distress among Indigenous Australian cancer survivors. Support Care Cancer. 2018;26(6):1737-46.

24.	Garvey G, Cunningham J, He VY, Janda M, Baade P, Sabesan S, et al. Health-related quality of life among Indigenous Australians diagnosed with cancer. Qual Life Res. 2016;25(8):1999-2008.

25.	Gillies M, Tan K, Anthony L, Miller F. Effect of Psychosocial, Behavioral, and Disease Characteristics on Health-Related Quality of Life (HRQoL) After Breast Cancer Surgery: A Cross-Sectional Study of a Regional Australian Population. Cureus. 2023;15(3):e36054.

26.	Hall A, Lynagh M, Tzelepis F, Paul C, Bryant J. How can we help haematological cancer survivors cope with the changes they experience as a result of their cancer? Ann Hematol. 2016;95(12):2065-76.

27.	Humpel N, Jones SC. Gaining insight into the what, why and where of complementary and alternative medicine use by cancer patients and survivors. Eur J Cancer Care (Engl). 2006;15(4):362-8.

28.	Johnston EA, Ekberg S, Jennings B, Jagasia N, van der Pols JC, Webb PM. Dietary Practices After Primary Treatment for Ovarian Cancer: A Qualitative Analysis From the OPAL Study. J Acad Nutr Diet. 2022;122(9):1607-28.e12.

29.	Jones C, Chapman YB. The lived experience of seven people treated with autologous bone marrow/peripheral blood stem cell transplant. Int J Nurs Pract. 2000;6(3):153-9.

30.	Joshy G, Khalatbari-Soltani S, Soga K, Butow P, Laidsaar-Powell R, Koczwara B, et al. Pain and its interference with daily living in relation to cancer: a comparative population-based study of 16,053 cancer survivors and 106,345 people without cancer. BMC Cancer. 2023;23(1):774.

31.	Kirby E, Kenny K, Broom A, Lwin Z. Chronicity in/and cancer: a qualitative interview study of health professionals, patients, and family carers. Crit Public Health. 2022;32(4):472-84.

32.	Kirby ER, Kenny KE, Broom AF, Oliffe JL, Lewis S, Wyld DK, et al. Responses to a cancer diagnosis: a qualitative patient-centred interview study. Supportive Care in Cancer. 2020;28(1):229-38.

33.	Lai-Kwon J, Khoo C, Lo S, Milne D, Mohamed M, Raleigh J, et al. The survivorship experience for patients with metastatic melanoma on immune checkpoint and BRAF-MEK inhibitors. J Cancer Surviv. 2019;13(4):503-11.

34.	Lai-Kwon J, Heynemann S, Flore J, Dhillon H, Duffy M, Burke J, et al. Living with and beyond metastatic non-small cell lung cancer: the survivorship experience for people treated with immunotherapy or targeted therapy. J Cancer Surviv. 2021;15(3):392-7.

35.	Lewis S, Broom A, Kenny K, Kirby E. Cancer caregivers' experiences of prognosis in Australia: a qualitative interview study. Bmj Open. 2020;10(1).

36.	Lewis S, Newton G, Kenny K, Boyle F. The incurable self: Negotiating social bonds and dis/connection with metastatic breast cancer. Sociol Health Illn. 2024;46(2):295-314.

37.	Lim CYS, Laidsaar-Powell RC, Young JM, Steffens D, Ansari N, Joshy G, et al. Healthcare experiences of people with advanced colorectal cancer: A qualitative study. Eur J Oncol Nurs. 2023;63:102265.

38.	Liu WH, Fox J, Yates P. Work-related experiences of prostate cancer survivors in Australia: a qualitative study. BMC Public Health. 2023;23(1):1806.

39.	Lisy K, Lai-Kwon J, Ward A, Sandhu S, Kasparian NA, Winstanley J, et al. Patient-reported outcomes in melanoma survivors at 1, 3 and 5 years post-diagnosis: a population-based cross-sectional study. Quality of Life Research. 2020;29(8):2021-7.

40.	Lynagh MC, Clinton-McHarg T, Hall A, Sanson-Fisher R, Stevenson W, Tiley C, et al. Are Australian clinicians monitoring medication adherence in hematological cancer survivors? Two cross-sectional studies. Exp Hematol Oncol. 2015;4.

41.	Lynch FA, Katona L, Jefford M, Ben Smith A, Shaw J, Dhillon HM, et al. Feasibility and Acceptability of Fear-Less: A Stepped-Care Program to Manage Fear of Cancer Recurrence in People with Metastatic Melanoma. J Clin Med. 2020;9(9).

42.	McGrath P. Normalcy: An Important Focus for Individuals Diagnosed with a Haematological Malignancy. Illness, Crisis & Loss. 2014;22(2):115-26.

43.	McGrath P, Holewa H. Patient stories: an innovative direction for nurses providing support to hematology patients in rural areas. Nurs Forum. 2014;49(3):182-8.

44.	McGrath P, Joske D, Bouwman M. Benefits from participation in the chemo club: psychosocial insights on an exercise program for cancer patients. J Psychosoc Oncol. 2011;29(1):103-19.

45.	Morris BA, Campbell M, Dwyer M, Dunn J, Chambers SK. Survivor identity and post-traumatic growth after participating in challenge-based peer-support programmes. Brit J Health Psych. 2011;16:660-74.

46.	Muhandiramge J, Zalcberg JR, Warner ET, Polekhina G, Gibbs P, van Londen GJ, et al. Cardiovascular disease and stroke following cancer and cancer treatment in older adults. Cancer. 2024;130(23):4138-48.

47.	Ng HS, Koczwara B, Roder D, Chan RJ, Vitry A. Patterns of health service utilisation among the Australian population with cancer compared with the general population. Aust Health Rev. 2020;44(3):470-9.

48.	O'Shaughnessy PK, Laws TA, Esterman AJ. Love, faith and hope - a secondary analysis of prostate cancer survivors and their partners. Contemp Nurse. 2015;50(2-3):149-68.

49.	Oberoi D, White V, Seymour J, Prince HM, Harrison S, Jefford M, et al. The course of anxiety, depression and unmet needs in survivors of diffuse large B cell lymphoma and multiple myeloma in the early survivorship period. J Cancer Surviv. 2017;11(3):329-38.

50.	Parton C, Ussher JM, Perz J. Hope, burden or risk: a discourse analytic study of the construction and experience of fertility preservation in the context of cancer. Psychol Health. 2019;34(4):456-77.

51.	Pascal J, Endacott R. Ethical and existential challenges associated with a cancer diagnosis. J Med Ethics. 2010;36(5):279-83.

52.	Pascal J, Johnson N, Dickson-Swift V, McGrath P, Dangerfield F. Understanding receptivity to informal supportive cancer care in regional and rural Australia: a Heideggerian analysis. Eur J Cancer Care. 2016;25(3):381-90.

53.	Paul CL, Hall AE, Carey ML, Cameron EC, Clinton-McHarg T. Access to Care and Impacts of Cancer on Daily Life: Do They Differ for Metropolitan Versus Regional Hematological Cancer Survivors? J Rural Health. 2013;29:S43-S50.

54.	Plage S. Deploying Normality: Cancer Survivor Identity and Authenticity in Ritual-like Practice. Medical Anthropology. 2021;40(5):473-89.

55.	Plage S. Cancer survivorship reimagined: A qualitative study on evolving interpretative repertoires of cancer and survivorship using participant-produced photography. Eur J Cancer Care. 2020;29(6).

56.	Plage S, Kirby E. Reconfiguring time: optimisation and authenticity in accounts of people surviving with advanced cancer. Health Sociol Rev. 2022;31(1):96-111.

57.	Player L, Mackenzie L, Willis K, Loh SY. Women's experiences of cognitive changes or 'chemobrain' following treatment for breast cancer: A role for occupational therapy? Aust Occup Ther J. 2014;61(4):230-40.

58.	Prior D. The meaning of cancer for Australian Aboriginal women; changing the focus of cancer nursing. Eur J Oncol Nurs. 2009;13(4):280-6.

59.	Pye S, Webster E, Zielinski R, Honeyball F. 'The best thing since sliced bread': Patient experiences of teleoncology in western NSW. Aust J Rural Health. 2023;31(1):90-7.

60.	Sharpe L, Butow P, Smith C, McConnell D, Clarke S. The relationship between available support, unmet needs and caregiver burden in patients with advanced cancer and their carers. Psycho-Oncology. 2005;14(2):102-14.

61.	Shaw LK, Sherman KA, Fitness J, Elder E, Australia BCN. Factors associated with romantic relationship formation difficulties in women with breast cancer. Psycho-Oncology. 2018;27(4):1270-6.

62.	Skandarajah AR, Lisy K, Ward A, Bishop J, Lacey K, Mann B, et al. Patient-reported outcomes in survivors of breast cancer one, three, and five years post-diagnosis: a cancer registry-based feasibility study. Quality of Life Research. 2021;30(2):385-94.

63.	Smith DP, Calopedos R, Bang A, Yu XQ, Egger S, Chambers S, et al. Increased risk of suicide in New South Wales men with prostate cancer: Analysis of linked population-wide data. Plos One. 2018;13(6).

64.	Stephens M, Halcomb E, Dewing J. Living on: An exploration of healthful cancer survivorship among grey nomads. The Australian Journal of Cancer Nursing. 2018;19(1):19-24.

65.	Stephens M, McKenzie H, Jordens CFC. The work of living with a rare cancer: multiple myeloma. J Adv Nurs. 2014;70(12):2800-9.

66.	Thandrayen J, Joshy G, Stubbs J, Bailey L, Butow P, Koczwara B, et al. Workforce participation in relation to cancer diagnosis, type and stage: Australian population-based study of 163,556 middle-aged people. J Cancer Surviv. 2022;16(2):461-73.

67.	Tran B, Vajdic CM, Webber K, Laaksonen MA, Stavrou EP, Tiller K, et al. Self-reported health, lifestyle and social circumstances of Australian adult cancer survivors: A propensity score weighted cross-sectional study. Cancer Epidemiol. 2020;67.

68.	Tzelepis F, Paul CL, Sanson-Fisher RW, Campbell HS, Bradstock K, Carey ML, et al. Unmet supportive care needs of haematological cancer survivors: rural versus urban residents. Ann Hematol. 2018;97(7):1283-92.

69.	Webb K, Sharpe L, Russell H, Shaw J. Fear of cancer recurrence in ovarian cancer caregivers: A qualitative study. Psychooncology. 2024;33(1):e6255.

70.	Yee J, Davis GM, Beith JM, Wilcken N, Currow D, Emery J, et al. Physical activity and fitness in women with metastatic breast cancer. J Cancer Surviv. 2014;8(4):647-56.

71.	Zhang YH, Joshy G, Glass K, Banks E. Physical functional limitations and psychological distress in people with and without colorectal cancer: findings from a large Australian study. J Cancer Surviv. 2020;14(6):894-905.

72.	Zopf EM, Newton RU, Taaffe DR, Spry N, Cormie P, Joseph D, et al. Associations between aerobic exercise levels and physical and mental health outcomes in men with bone metastatic prostate cancer: a cross-sectional investigation. Eur J Cancer Care. 2017;26(6).
